# Associations of CAIDE dementia risk score with glucose and lipid‐related metabolic blood biomarkers in the FINGER study participants

**DOI:** 10.1002/alz.092328

**Published:** 2025-01-09

**Authors:** Ana Sabsil Lopez Rocha, Ruth Stephen, Alina Solomon, Tiia Ngandu, Jenni Lehtisalo, Tiina Laatikainen, Patrizia Mecocci, Jaakko Tuomilehto, Miia Kivipelto, Francesca Mangialasche

**Affiliations:** ^1^ Division of Clinical Geriatrics, Center for Alzheimer Research, Department of Neurobiology, Care Sciences and Society (NVS), Karolinska Institute, Stockholm Sweden; ^2^ University of Eastern Finland, Kuopio Finland; ^3^ Division of Clinical Geriatric (NVS), Karolinska Institutet, Sweden, Stockholm Sweden; ^4^ Department of Clinical Medicine/Neurology, University of Eastern Finland, Kuopio, Finland, Kuopio Finland; ^5^ Population Health Unit, Finnish Institute for Health and Welfare, Helsinki Finland; ^6^ Population Health Unit Finnish Institute for Health and Welfare, Helsinki Finland; ^7^ Institute of Public Health and Clinical Nutrition, University of Eastern Finland, Kuopio Finland; ^8^ Division of Clinical Geriatrics, NVS, Karolinska Institutet, Stockholm, Sweden, Stockholm Sweden; ^9^ Institute of Gerontology and Geriatrics, Department of Medicine, University of Perugia, Perugia Italy; ^10^ Department of Public Health, University of Helsinki, Helsinki Finland; ^11^ Diabetes Research Unit, King Abdulaziz University, Jeddah Saudi Arabia; ^12^ Population Health Unit, Finnish Institute for Health and Welfare, Helsinki Finland; ^13^ South Ostrobothnia Central Hospital, Seinäjoki Finland; ^14^ Institute of Public Health, University of Eastern Finland, Kuopio Finland; ^15^ The Ageing Epidemiology (AGE) Research Unit, School of Public Health, Imperial College London, London United Kingdom; ^16^ Division of Clinical Geriatrics, Center for Alzheimer Research, Department of Neurobiology, Care Sciences and Society, Karolinska Institutet, Stockholm Sweden; ^17^ Karolinska University Hospital, Stockholm Sweden; ^18^ Division of clinical Geriatrics, Department of Neurobiology, Care Sciences and Society, Karolinska Institutet and Stockholm University, Stockholm Sweden; ^19^ Karolinska University Hospital, Theme Inflammation and Aging, Solna Sweden

## Abstract

**Background:**

The Cardiovascular risk factors, aging, and dementia (CAIDE) risk score is a validated tool estimating dementia risk. We investigated the association of CAIDE score with 12 markers of glucose and lipid metabolism, in the Finnish Geriatric Intervention Study to Prevent Cognitive Impairment and Disability (FINGER) participants.

**Methods:**

The FINGER trial had 1260 participants, aged 60–77 years, with a CAIDE score ≥6, without substantial cognitive impairment. At baseline, a panel of 12 glucose and lipid metabolism blood markers (adiponectin, adipsin, c‐peptide, ghrelin, gastric inhibitor polypeptide (GIP), glucagon‐like‐peptide (GLP‐1), glucagon, insulin, leptin, plasminogen activator in plasma (PAI‐1), resistin, and visfatin) were measured using Bio‐Plex‐200. CAIDE dementia risk score (age, sex, education, blood pressure, body mass index (BMI), cholesterol, physical activity) was also available. Linear regression models were fit using CAIDE score as the outcome, metabolic markers as predictors and APOE4 as covariate. We also tested the interactions between the metabolic markers and APOE4.

**Results:**

Participants with the metabolic markers (n=813) were younger (p=0.01), had lower levels of HDL (p=0.01) and higher levels of systolic blood pressure (p= 0.02), HbA1c (p=0.00), BMI (p=0.00), CRP (p=0.02) and higher CAIDE risk score (p=0.02) but slightly better memory (p=0.00) compared to those without the metabolic markers (n=447).

Initially, CAIDE score was tested with each metabolic marker separately. Interactions between metabolic markers and APOE4 were also tested. Higher CAIDE score was associated with lower adiponectin (β coefficient= ‐0.01, p=0.01), ghrelin (β = ‐0.13, p<0.001), and higher insulin (β =0.16, p<0.001), leptin (β=0.15 p<0.001), PAI‐1 (β =0.14, p<0.001) and c‐peptide (β=0.15 p<0.001). No interactions between metabolic markers and APOE were significant except for glucagon x APOE4 (β= ‐2.10, p=0.021).

Next, the metabolic markers with the significant associations were added to a mutually adjusted model. The associations of CAIDE score with ghrelin (β= ‐0.22, p<0.001) and insulin (β=0.124, p=0.03) remained significant.

**Conclusion:**

Increased dementia risk in older people is linked to glucose and lipid imbalance. The identification of phenotypes through different metabolic markers and the role of genetic factors on these metabolic markers can lead to a better stratification of the population at risk of developing dementia.

